# A Practical Treatise on the Special Diseases of the Skin

**Published:** 1845-10

**Authors:** 


Art. V.—*A Practical Treatise on the Special Diseases of the Skin.* By C. M. Gibert, Physician to the Hôpital St. Louis, &c. Translated from the French by Edgar Sheppard.—*London*, 1845. 8vo, pp. 362.

The work of Gibert on the diseases of the skin differs from all other treatises on the same subject, with the exception of Plumbe's, in being confined to the consideration of the special and more common cutaneous affections which fall under the daily observation of every practitioner. No mention is made in it of the contagious exanthemata, of the varioloid diseases, of those that are peculiar to other countries, that are very rare in the west of Europe, or about which much doubt and uncertainty prevail.

The original of the volume before us has been long known to all dermatologists as a sufficiently practical and useful work on the subject of which it treats. Its distinguishing feature consists in the fulness of its therapeutic details. Indeed, these are at times too copious; the author being apt to confuse his readers with particulars about the preparation of decoctions, ointments, &c., that would have been more in place in an appendix than in the body of the book.

Allowing all praise, however, to Gibert's work, the expediency of translating it may, we think, very fairly be questioned. As a practical treatise, it is most decidedly not equal to the work of Messrs. Cazenave and Schedel, lately translated by Dr. Burgess, to which it is very much inferior in style and arrangement, and in the amount of information given on several very important subjects, as, for instance, the syphilitic diseases of the skin and lupus. As a work of learning and research, it falls short of Willan's treatise, and of Bateman's synopsis; and in neither respect is it at all equal to the classical treatise by Rayer, which was a few years since put into an

English garb by Dr. Willis. The expediency of undertaking the translation, however, is perhaps rather a question between Mr. Sheppard and his publisher, than between him and us; the manner and style in which it has been executed concern us more nearly; and in this respect Mr. Sheppard has done but scanty justice to his original, and but little credit to himself. Indeed, he appears to have had some misgivings on this point, for he apologises in the preface for the want of elegance and the literal character of his version, and for his not having rendered it more free from gallicisms. With these, indeed, every page of the book abounds: thus we find Plato, Prosper Alpinus, and the Ephemerides Germanicæ, Frenchified into *Platon*, Prosper *Alpin*, the Ephemerides *Germaniques;* and such expressions as the following meet the eye at every turn: "The frequency of erections reacting *sur le moral.*" "A *profound* alteration of the blood and lymph;" "the evil apparent is always indicative;" "a veritable fluxion;" "this often so fatal disease;" "the breath of life which yet animated him was snapped." Passages of this description we might multiply to an endless extent; we have, however, given enough to justify our criticism.

Occasionally Mr. Sheppard fails in conveying the author's meaning, or at all events, makes it somewhat difficult for the reader to guess. Thus, for instance, at p. 97, we find the following sentence: "Sanguineous and lymphatic subjects are most affected with it (scabies), but we must observe that in France this is the most general temperament, and that it would be unfair uniformly to attribute this fact to a greater natural predisposition to contagious diseases, and to a more active absorption really existing in this species of temperament." In this passage "*this fact*" refers to the sanguineous and lymphatic temperaments being the most common in France, and not, as the author intends it, to persons of those temperaments being the most readily affected with scabies. Again at p. 356, we find the following very obscure and ungrammatical sentence: "Syphilis in the newborn infant is always a severe malady; *it* frequently falls a victim to *it* in a few weeks; nevertheless if *it* is well constituted," &c.—leaving us in doubt whether syphilis falls a victim to the infant, or the infant to the disease.

Should a second edition of this work ever be called for,—which in the present overstocked state of the literature of the diseases of the skin we much doubt,—we trust that Mr. Sheppard will take the trouble carefully to revise the style of his translation, the character of which, materially lessens the value of a sufficiently useful manual;—rendering it very doubtful, to use his own poetical expression, whether it "will live as long, bearing the union jack of England, as the tricoloured flag of France."

We have been the more desirous of calling attention to the very imperfect style of this volume, because having criticised somewhat severely in other pages of our present Number, some American translations of French works, we wish to prove to our transatlantic friends, that our justice is evenhanded and without respect of kin or country. *Tros Tyriusve,* has ever been the motto of our Journal.

